# Validation of a Duplex Digital PCR Assay for the Quantification of the NK603 Maize Event Across Three dPCR Platforms

**DOI:** 10.3390/foods15081366

**Published:** 2026-04-14

**Authors:** Daniela Verginelli, Katia Spinella, Sara Ciuffa, Raffaele Carrano, Davide La Rocca, Elisa Pierboni, Monica Borghi, Silvana Farneti, Ugo Marchesi

**Affiliations:** 1 National Reference Laboratory for GM Food and Feed, GMO Unit, Istituto Zooprofilattico Sperimentale del Lazio e della Toscana “Mariano Aleandri”, 00178 Rome, Italy; daniela.verginelli@izslt.it (D.V.); sara.ciuffa@izslt.it (S.C.); raffaele.carrano-esterno@izslt.it (R.C.); davide.larocca@izslt.it (D.L.R.); ugo.marchesi@izslt.it (U.M.); 2 Istituto Zooprofilattico Sperimentale dell’Umbria e delle Marche “Togo Rosati”, 06126 Perugia, Italy; e.pierboni@izsum.it (E.P.); m.borghi@izsum.it (M.B.); s.farneti@izsum.it (S.F.)

**Keywords:** validation study, digital PCR, GMO

## Abstract

In the European Union, mandatory labeling of food and feed products is required when authorized genetically modified organisms (GMOs) exceed 0.9% per ingredient, necessitating reliable analytical methods for official control laboratories. Event-specific PCR assays validated according to ISO/IEC 17025 are the reference approach for GMO detection, identification, and quantification. The growing use of digital PCR (dPCR) has encouraged the adaptation of real-time PCR methods to dPCR-based strategies, as dPCR enables absolute quantification without calibration standards, shows reduced sensitivity to inhibitors, and allows for the design of a multiplex assay. In this study, an *in-house* validation of a duplex dPCR assay targeting the maize GM event NK603 and the HMG reference gene was performed on three platforms: Bio-Rad QX200™ (Pleasanton, CA, USA), Qiagen QIAcuity (Venlo, The Netherlands), and Thermo Fisher QuantStudio Absolute Q (Waltham, MA, USA). All validation parameters met the Joint Research Centre (JRC) acceptance criteria. In particular, this assay demonstrated high specificity, sensitivity (limit of quantification or LOQ < 35 copies per reaction), precision, and trueness (RSDr and bias < 25%). The data indicate that the duplex dPCR assay can be used for routine GMO analysis and future collaborative validation studies.

## 1. Introduction

The second most widely planted genetically modified (GM) crop, after soybean, is maize with 68.4 million hectares, having a rate of global adoption of 33.7% as reported by the AGBIOS 2024 review [1].

Moreover, the ever-increasing number of GM events raises additional challenges for the official control of genetically modified organisms (GMOs) and for policy-making.

To support informed consumer choice, GM labeling policies have been adopted worldwide, although threshold levels vary considerably: in the European Union, mandatory labeling is required for foods with more than 0.9% GM material per ingredient [2]; in Australia and New Zealand, labeling is required above 1% GM material; in the Republic of Korea and China, the threshold is 3%; in Japan, designated foods with 5% or more GM material by weight must be labeled and in the United States, mandatory “bioengineered” labeling allows up to 5% inadvertent GM presence per ingredient; and Switzerland sets a 3% threshold for raw materials or feeds containing only one GM ingredient, and a 2% threshold per ingredient for feeds with multiple GM ingredients [3].

As a matter of fact, the use of reliable analytical methods adopted by official control laboratories is crucial. The gold standard is represented by quantitative real-time PCR (qPCR); nevertheless, it is subject to limitations, for instance, when quantifying a large number of GMO targets within a single sample [4]. On the other hand, digital PCR (dPCR) is a sensitive nucleic acid quantification PCR technology, in which individual partitions are measured independently based on their endpoint fluorescence reporting a binary outcome standing for presence or absence for the target [5]. Some of the advantages are its absolute quantification with no standard curves, mitigating the impact of inhibitors, and precision of quantification that performs better than qPCR [6,7,8,9,10,11]. However, the dynamic range of dPCR is inherently constrained by the total partition count [10]. So far, a high number of qPCR assays has been transformed to dPCR [11,12,13,14,15,16,17,18,19,20,21,22].

Besides, dPCR platforms using different technologies are available, including droplet digital PCR (ddPCR), nanoplate dPCR, and chamber or chip-based dPCR (cdPCR). In ddPCR, all reagents are dispersed in nanoliter-sized droplets (e.g., Bio-Rad QX200™ ddPCR system) and each droplet, which equals to a PCR reaction, is obtained in a water–oil emulsion to form partitions that separate template DNA molecules [23]. In nanoplate dPCR (e.g., Qiagen QIAcuity One system), a dedicated plate is needed for the partitioning step. This system integrates all the steps (partitioning, thermocycling, and imaging) into a single dPCR instrument [24,25] and the results are expressed in copies of the target sequence per microliter of reaction. Eventually, cdPCR (e.g., QuantStudio Absolute Q Digital PCR System Thermo Fisher Scientific) reduces the workflow by eliminating droplet generation and emulsification steps. It is composed of a pre-defined microchamber structure, which ensures uniform partition volumes and better thermal uniformity, and minimizes cross-contamination risks. Its support image-based fluorescence detection allows us to obtain a tangible precise duplex or multiplex quantification reducing the signal overlap [21,26].

The aim of this study was to perform an *in-house* validation of a quantitative duplex dPCR assay targeting GM event NK603 maize (Unique identifier MON-ØØ6Ø3-6 authorized in European Union) and the high-mobility-group (HMG) reference gene, comparing three different dPCR platforms. The European Union Reference Laboratory for Genetically Modified Food and Feed (EURL-GMFF) has validated the event-specific qPCR method for genetically modified food and feed [27,28,29,30]. The identical primer–probe set was used for the three platforms, and the optimization of the reaction conditions and method verification were performed according to the published guidelines, including adjustments to primer and probe concentrations. Particularly, dPCR performance was assessed taking into account the specificity, cross-talk, asymmetric limit of quantification (LOQasym), robustness, dynamic range, linearity, and accuracy (trueness and precision) parameters [5,31,32,33,34,35]. In addition, the measurement uncertainty (MU) was evaluated as reported in the ENGL document [36,37]. Although several studies have focused on the transfer of qPCR methods to digital PCR, comparisons of dPCR platforms using different partitioning principles within a single laboratory based on identical samples and harmonized validation procedures remain limited.

In this context, the present study provides a comprehensive cross-platform evaluation of a duplex dPCR assay for NK603 quantification under controlled and comparable experimental conditions.

## 2. Materials and Methods

### 2.1. DNA Extraction and Evaluation of DNA Quality

The NK603 MAIZE ERM-BF415f (49.1 g/kg), ERM-BF415e (19.6 g/kg), ERM-BF415d (9.8 g/kg), ERM-BF415c (4.9 g/kg), ERM-BF415b (1 g/kg), and non-modified maize ERM-BF415a (<0.4 g/kg) CRMs were purchased from the Joint Research Centre (JRC) of the European Commission (Geel, Belgium). DNA extraction starting from 200 mg of CRMs was performed using the Maxwell^®^ RSC PureFood GMO extraction method with the Maxwell^®^ RSC Instrument (Promega Corporation, Madison, WI, USA), according to the manufacturer’s instructions, for the Bio-Rad and Qiagen platforms. For the Thermo Fisher platform, DNA was extracted using a method based on a 2% CTAB buffer, as described in ISO 21571:2013/Amd 1:2013 [38].

DNA quality was verified by diluting the extracted DNA in nuclease-free water (Sigma-Aldrich Chemie GmbH, Munich, Germany) and analyzing it by dPCR using an endogenous reference gene (HMG), as described in the JRC Technical Report 2023 [39]. A five-point inhibition test was performed, in which the average absolute copy number per reaction measured in the diluted samples, multiplied by the corresponding dilution factor, was required not to differ by more than 25% from the average absolute copy number per reaction measured at the highest DNA concentration. All extracted DNA samples were then stored at +4 °C.

### 2.2. Sample Preparation

**Figure 1:**
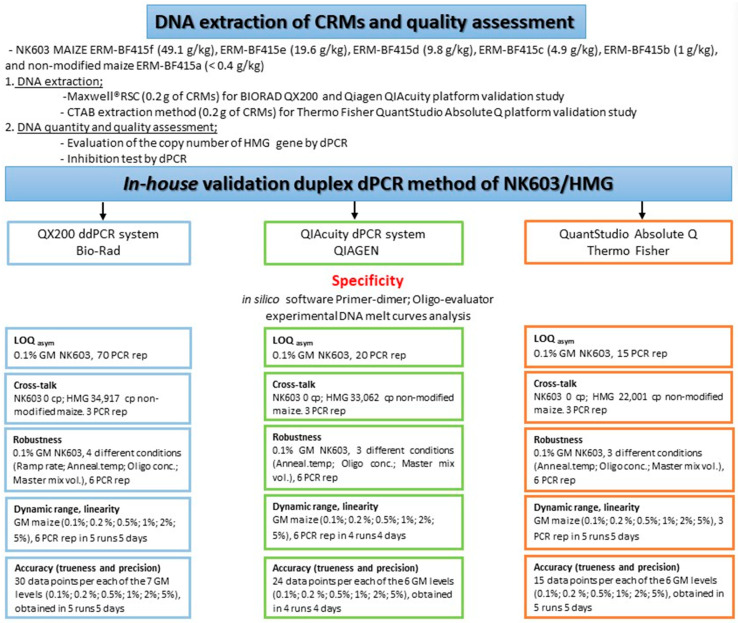
Workflow and *in-house* validation of the NK603/HMG duplex dPCR method across three platforms (Bio-Rad QX200™, QIAGEN QIAcuity, and Thermo Fisher QuantStudio Absolute Q), including DNA extraction, DNA quality assessment, and evaluation of validation parameters.

The validation study was designed to evaluate six different GM mass fractions (% *m*/*m*) (see Figure 1). Since the 0.2% *m*/*m* certified reference material was not commercially available, this selected GM concentration was obtained by preparing *in-house* mixtures of GM-positive material with corresponding non-GM material according to the JRC Technical Report [37,39]. The preparation of the mixture was based on the absolute copy number of the HMG previously determined by digital PCR, in order to ensure accurate target quantification. The mixture was prepared under controlled conditions to guarantee homogeneity and consistency of the GM content, and was subsequently used for the validation of the digital PCR assays.

### 2.3. Workflow and Data Analysis of dPCR Analysis

The *in-house* validation of the genetically modified maize event NK603 was performed using three digital PCR (dPCR) platforms: the QX200™ Droplet Digital PCR (ddPCR) (Bio-Rad Laboratories, Pleasanton, CA, USA), the QIAcuity Digital PCR System (QIAGEN, Hilden, Germany), and the QuantStudio Absolute Q Digital PCR System (Thermo Fisher Scientific, Waltham, MA, USA) [23,40,41] (Figure 1).

The NK603 real-time PCR assay, previously validated by EURL-GMFF, was subsequently optimized and applied across the different platforms to allow for methodological comparison.

The *in-house* validation study was performed in accordance with the requirements set out in the JRC guidance documents [5,31,35] and ISO 20395 [34]. The following performance parameters were evaluated: specificity, cross-talk, asymmetric limit of quantification (LOQasym), robustness, dynamic range, linearity, accuracy (trueness and precision), and measurement uncertainty (MU). The experimental design and analytical workflow adopted for each validation parameter are summarized in Figure 1. Specificity of the NK603/HMG duplex assay was first assessed in silico using Primer-Dimer software (version 2018) [42] to predict potential oligonucleotide hybridization events and OligoEvaluator software (version 2014) [43] to evaluate cross-dimer and hairpin formation. Experimental evaluation of specificity was performed through DNA melting curve analysis, which confirmed the amplification of a single, specific product. These analyses were performed on a Rotor-Gene Q 5plex system (Qiagen GmbH, Hilden, Germany) using a 20 μL reaction volume containing 1× FAST Eva Green qPCR Master Mix (Fisher Molecular Biology, Rome, Italy), 1 ng of genomic DNA, and 150 nM of each forward and reverse primer. Each sample was analyzed in duplicate. Thermal cycling consisted of initial denaturation at 95 °C for 2 min, followed by 40 cycles of 95 °C for 5 s, 60 °C for 30 s, and 72 °C for 20 s, with fluorescence acquisition during the annealing step. Melting curve analysis was carried out using temperature increments of 0.1 °C with a 30 s hold at each step. Cross-talk was measured in terms of fluorescence signal in the presence of an excess of the HMG gene and in the absence of a GM NK603 maize event as shown in Figure 1.

Sensitivity was assessed at the 0.1% *m*/*m* GM level, representing low NK603 content against a high HMG background under asymmetric conditions, with the number of replicates adapted to the specific digital PCR platform, according to the plate and the corresponding number of available wells [32].

Robustness testing was conducted at the 0.1% *m*/*m* GM level as described on the JRC technical report [32]. When creating a multifactorial experiment design on the QX200™ platform, four variations were applied: (i) a 10% reduction in reaction volume, (ii) an increase of 1 °C in annealing temperature, (iii) a slower ramp rate (+0.5 °C/s), and (iv) a 10% decrease in primer and probe concentrations. Due to platform-specific limitations, robustness testing on the QIAcuity and Thermo Fisher system included only three variations: (i) a 10% reduction in reaction volume, (ii) an increase of 1 °C in annealing temperature, and (iii) a 10% reduction in oligonucleotide concentrations. The introduced modifications and the assay performances were compared across three selected dPCR platforms. Method performance was assessed in terms of mean measured GMO content (% *m*/*m*), repeatability (RSDr %), and trueness (bias %).

The dynamic range and the linearity of the duplex digital PCR assay targeting the HMG reference gene and the NK603 maize event were evaluated using CRMs with GM mass fractions ranging from 0.1% to 4.91% (*m*/*m*).

Accuracy (trueness and precision) were calculated as RSDr% and Bias % at each tested % *m*/*m* level in different PCR replicates depending on the platform obtaining different data points. For the Bio-Rad QX200™ platform, six replicates at each GM NK603 level were analyzed across five independent runs performed over five consecutive days. On the QIAcuity platform, six replicates per GM level (% *m*/*m*) of NK603 were analyzed in four runs conducted over four days. On the Thermo Fisher platform, three replicates per GM level (% *m*/*m*) of NK603 were analyzed in five runs conducted over five days.

The presence of intermediate fluorescence signals (“rain”) was also evaluated using the approach described by Lievens et al. [44]. In accordance with Pecoraro et al. [5], the proportion of rain was required to be below 2.5% of the total number of partitions.

### 2.4. Optimization of dPCR Conditions

PCR was performed based on officially validated qPCR protocols for maize GM event NK603 [28] in combination with the high-mobility group (HMG) reference gene [29] with some modifications (Table S1). As a matter of fact, the HMG reference gene is considered to be more stable then alcohol dehydrogenase 1 (ADH1) in terms of quantification performance according to Paternò et al. (2009) and Jacchia et al. (2018) [45,46].

Primer and probe concentrations were subsequently optimized, and the final concentrations are reported in Table S1.

Figure S1 shows the optimization of primer and probe concentrations for NK603. Reactions were tested using low (0.15 pmol/µL primers, 0.05 pmol/µL probe; left panels) and high (0.6 pmol/µL primers, 0.25 pmol/µL probe; right panels) oligonucleotide concentrations. The low primer and probe concentrations correspond to the original real-time PCR method validated by JRC [28,30], whereas increased concentrations (0.6 pmol/µL primers and 0.25 pmol/µL probe) were evaluated for digital PCR optimization. Each condition was tested at three target levels (0.1%, 1%, and 5% *m*/*m*). Higher primer and probe concentrations led to increased fluorescence amplitudes and improved cluster separation for both targets.

For the QX200™ and QuantStudio Absolute Q dPCR platforms, optimization was performed only for the transgene, as the endogenous target already showed a clear signal with well-separated positive and negative droplet clusters under the initial conditions. For the QIAcuity platform, the primer and probe concentrations recommended by the manufacturer were used. Since these conditions provided a strong fluorescence signal and clear cluster separation, no further optimization was carried out.

### 2.5. QX200™ ddPCR System

Analyses performed on the QX200™ ddPCR system followed a droplet-based workflow. Droplet digital PCR analyses were carried out using a QX200™ ddPCR system (Bio-Rad, Pleasanton, CA, USA). Reactions were prepared in a final volume of 20 μL comprising 1× ddPCR Supermix for probes (no dUTP) (Bio-Rad), 4 μL of DNA template, and primers and probes at the concentrations indicated in Table S1. Droplet generation was performed using DG8 cartridges loaded into a QX200™ droplet generator (Bio-Rad). The resulting emulsions were transferred to 96-well plates and amplified using a Veriti™ 96-Well Fast Thermal Cycler (Thermo Fisher Scientific, Waltham, MA, USA). The thermal cycling protocol included an initial enzyme activation step at 95 °C for 10 min, followed by 45 cycles consisting of denaturation at 95 °C for 30 s and combined annealing/extension at 60 °C for 60 s, with a final droplet stabilization step at 98 °C for 10 min and a hold at 4 °C. After amplification, droplets were read using a QX200™ droplet reader (Bio-Rad), and fluorescence data were processed with QX Manager software version 2.1 Standard Edition (Bio-Rad). Only results meeting predefined quality criteria were considered for further analysis, including a single, well-defined positive droplet population, more than 10,000 accepted droplets per well in a 20 μL reaction, clear separation between positive and negative droplets, no more than two positive droplets in the no-template control, and more than two positive droplets in positive controls and samples [23,47]. Target concentrations were ultimately reported as copies of the target sequence per microliter of reaction.

### 2.6. QIAcuity dPCR System

Digital PCR analyses on the QIAcuity platform were conducted using a fully integrated system in which partitioning, thermocycling, and fluorescence imaging were performed on a single instrument. Reaction mixtures were prepared according to the manufacturer’s instructions and loaded onto QIAcuity Nanoplate 26K microfluidic plates, providing 24 reaction wells with approximately 26,000 partitions per well. Quantification of the NK603 event was performed using a QIAcuity One digital PCR system (Qiagen). Reactions were prepared with 4× Probe PCR Master Mix, genomic DNA as a template, primers at a final concentration of 800 nM, and probe value at 400 nM for the endogenous reference and transgene. The reaction mix containing the DNA template was loaded onto a 24-well QIAcuity Nanoplate 26k (Qiagen, Hilden, Germany) at a final volume of 40 μL per well, and the plate was sealed using the appropriate nanoplate sealing film provided by the manufacturer.

Thermal cycling consisted of an initial activation step at 95 °C for 2 min, followed by 40 amplification cycles comprising denaturation at 95 °C for 15 s and annealing at 60 °C for 30 s. Fluorescence signals were acquired and analyzed using QIAcuity Software Suite version 2.5.0.1 (Qiagen, Hilden, Germany), with image acquisition performed at an exposure time of 500 ms and a gain setting of 6. The same quality criteria applied to Bio-Rad QX200™ were used for result evaluation. Results are expressed as copies of the target sequence per microliter of reaction, together with the corresponding 95% confidence interval automatically calculated by the software.

### 2.7. QuantStudio Absolute Q Digital PCR System

Digital PCR analyses were performed using QuantStudio Absolute Q (Thermo Fisher Scientific), a plate system with automated partitioning of samples into 20,480 microchambers and integrated amplification and fluorescence detection (five optical channels with one, ROX™, dedicated to quality control). Duplex PCR performed on QuantStudio Absolute Q (Thermo Fisher Scientific) was carried out in a final volume of 10 µL, containing 2 µL of Absolute Q Universal DNA Digital PCR Master Mix (5×), 0.5 µL of the NK603 assay (20×), 0.5 µL of the HMG assay (20×), 2 µL of DNA template, and 5 µL of nuclease-free water.

Primers and probes were used at concentrations indicated in Table S1. Nine microliters of each PCR mixture were loaded onto each well of a MAP16 plate, partially reusable with a 4 × 4 well format, and 15 µL of isolation buffer was added. MAP plate gasket strips were placed on all columns, and the plate was loaded into the dPCR instrument. PCR amplification was carried out for 1 h 20 min under the following conditions: 96 °C for 10 min, followed by 40 cycles of 96 °C for 5 s and 60 °C for 15 s. Data acquisition and analysis were performed using QuantStudio Absolute Q Digital PCR software, version 6.3.5, with manual determination of quality thresholds (10,000 and 1000 for NK603 and HMG, respectively). Arrays with >20,210 passed microchambers per array were considered valid according to the manufacturer’s reference guide. Final target concentrations were expressed as copies of the target sequence per microliter of reaction, along with the corresponding 95% confidence intervals calculated automatically by the software. In all three platforms, a no-template control (NTC) was included in each run to monitor potential cross-contamination during plate preparation.

### 2.8. Calculation of GM Content

For each sample, copy numbers corresponding to both NK603 target HMG were determined using the digital PCR instrument’s software, and the GM content (% *m*/*m*) was calculated according to the formula reported in the application V 1.0 issued by the European Union Reference Laboratory for Genetically Modified Food and Feed (EURL-GMFF) [48].

For the NK603 event, the conversion factor (CF) was set to 0.51, as reported in version 12 of the table “Conversion Factors (CFs) for Certified Reference Materials (CRMs)” [49] published by the Joint Research Centre (JRC), which was the version available at the time this method validation study was conducted.

### 2.9. Measurement Uncertainty (MU)

The uncertainty associated with the determination of GM content was estimated by taking into account the main sources of variability affecting the measurement, including pipetting performance and, where applicable, droplet volume. The evaluation was carried out in accordance with the established guidance on measurement uncertainty in GMO analysis and with the European technical guidance for laboratories performing GMO quantification under a flexible scope of accreditation [36,37].

A detailed description of the calculation approach is provided by Verginelli et al. [20] and in Figure S2.

## 3. Results and Discussion

### 3.1. Specificity

The analysis focused on the prediction of secondary structure formation and on the evaluation of Gibbs free energy (ΔG) variations associated with potential oligonucleotide interactions. ΔG values lower than −9 kcal/mol are indicative of a high likelihood of primer–dimer formation. Given the multiplex nature of the assay, all possible combinations among NK603- and HMG-specific primers and probes were examined, as unintended amplification products may be present when complementary sequences are present in the target DNA. No relevant risk of dimer formation was identified for any oligonucleotide combination, although weak secondary structures were predicted in silico (Table S2).

Experimental confirmation of assay specificity was subsequently obtained through post-amplification melting curve analysis. The absence of additional melting peaks beyond those expected for the NK603 and HMG targets demonstrated that non-specific amplification products were not generated in the duplex reaction, thereby confirming the high specificity of both primer/probe systems (Figure S3).

### 3.2. Cross-Talk

The cross-talk assessment was performed using non-modified maize (ERM-BF415a) containing 34,917 HMG copies per reaction, analyzed in three replicates per condition on the Bio-Rad QX200™ system. A corresponding experiment on the QIAcuity platform was carried out with HMG 33,062 copies per reaction as well as on the Thermo Fisher platform with 22,001 HMG copies per reaction. In all three cases, no cross-talk signals were detected. According to the guidelines for the verification of methods using dPCR [33], this parameter was therefore deemed not relevant. Nevertheless, when employing uncommon fluorophore/quencher combinations, an optional evaluation of optical cross-talk may still be useful, depending on the fluorescence channel configuration of the instrument.

### 3.3. Sensitivity (LOQasym)

The mean GM copy number per reaction was 17.20 copies for the Bio-Rad system, 33.70 copies for the QIAcuity system, and 24.26 copies for the Thermo Fisher system, while the corresponding endogenous gene copy numbers were 33,311, 59,240 and 44,695 copies per reaction, respectively (Table 1).

The precision at the LOQ level was acceptable for all platforms, with RSDr ranging from 23.08% to 24.93%. A limited number of outliers was observed, with three for Bio-Rad, one for QIAcuity, and none for Thermo Fisher. These results demonstrate that the 0.1% GM level can be reliably quantified under asymmetric duplex conditions. The obtained performance parameters comply with the minimum performance requirements for quantitative GMO analysis [31,32,35].

### 3.4. Robustness

Under nominal conditions, with the annealing temperature and ramp rate unchanged, comparable mean NK603 contents were obtained across all platforms, ranging from 0.09% to 0.14% *m*/*m* (Table 2).

Reductions of 10% in primer/probe concentrations, master mix volume, or both resulted in only minor changes in mean values on all platforms. Although an increase in RSDr and bias was observed in some conditions, particularly on the Bio-Rad QX200™ system, measured GMO contents remained consistent with the original protocol. An increase in the annealing temperature by +1 °C did not substantially affect NK603 quantification.

Greater variability was observed when combining an increase in the annealing temperature (+1 °C) with ramp rate (+0.5 °C/s), as well when increasing the ramp rate alone. These conditions were assessed exclusively on the Bio-Rad QX200™ platform.

Under these stressed conditions, RSDr values reached up to 29.57%, while mean NK603 contents remained close to the target value (0.11–0.14%). Bias values ranged from 8.69% to 29.83%. Although these values indicate increased variability, they remain below the 30% threshold and therefore comply with the acceptance criteria established by the EURL [31,35] (Table 2) for low concentration levels (0.1% *m*/*m*), where higher variability is expected due to proximity to the quantification limit.

Comparable performance was observed across the three dPCR platforms under standard conditions, supporting the reliability and transferability of the method for routine GMO analysis.

### 3.5. Dynamic Range and Linearity

Across all the tested range, all platforms provided consistent quantification of the NK603 event and met the acceptance criteria for precision (RSDr ≤ 25%) and trueness (bias ≤ 25%), as shown in Table 3, demonstrating suitability for GMO percentage determination within the tested interval [31,35].

**Figure 2:**
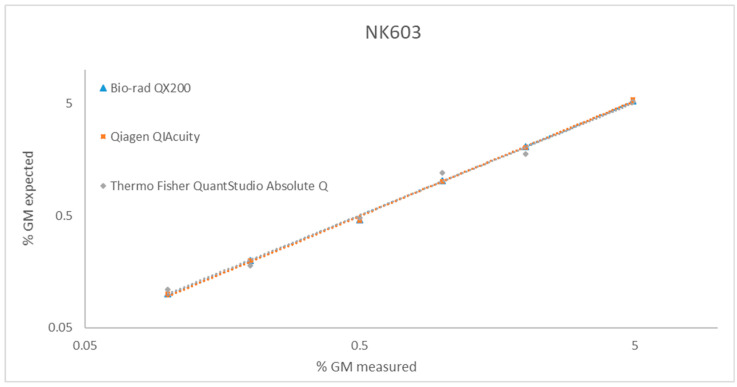
Correlation between measured and expected % GM for sample NK603 across three digital PCR platforms (Bio-Rad QX200™, QIAGEN QIAcuity, and Thermo Fisher QuantStudio Absolute Q).

The linearity was evaluated within the dynamic range and assessed by linear regression analysis of measured versus nominal GM levels over several independent PCR runs (Table 4 and Figure 2).

The high coefficients of determination (R^2^ ≥ 0.98) obtained across all platforms and PCR runs demonstrate excellent linearity of the duplex dPCR assay within the tested GM range. Minor differences in slope values among platforms did not affect the proportionality between measured and expected GM levels, confirming the robustness and transferability of the method for NK603 quantification across different dPCR technologies.

### 3.6. Trueness and Precision

Trueness was determined as the relative difference between the average measured concentration (% *m*/*m*) of the sample and the certified concentration. As shown in Table 5, the bias (%) between multiplex dPCR results and the certified values was below 25% for all GM levels and for all dPCR platforms tested, fully complying with the ENGL performance criteria for GMO analytical methods [31,35].

Overall, the comparison among platforms demonstrates good agreement in terms of trueness, with measured GM contents closely matching the certified values across the entire dynamic range (0.1–5% *m*/*m* GM). Both Bio-Rad QX200™ and Qiagen QIAcuity exhibited consistently low bias values, generally below 10%, confirming the high accuracy of droplet-based and nanowell-based digital PCR technologies. Thermo Fisher QuantStudio Absolute Q, despite employing a lower number of replicates, showed a trueness performance fully comparable to the other platforms, with bias percentages always remaining within the ENGL acceptance limits [31,35].

The precision was evaluated in terms of Repeatability Standard Deviation (RSDr). All platforms met the established criteria (Table 5) with an RSDr ≤ 25%, although slight differences were observed. Bio-Rad QX200™ showed consistent repeatability at intermediate GM levels, with slightly higher RSDr values in the range of 0.1–0.2% GM. As can be observed in Table 5, this method satisfies this requirement at all GM levels tested.

In addition to analytical performance, a systematic comparison of operational aspects, such as sample throughput, hands-on time, ease of use, data analysis and interpretation, instrument footprint, and maintenance requirements, may further support informed technology selection. The Bio-Rad QX200™ droplet digital PCR platform, based on droplet generation and subsequent reading steps, involves a multi-step workflow with separate instruments, which may increase the hands-on time, but offers flexibility within standard 96-well plate formats. In contrast, the QIAcuity Digital PCR System and QuantStudio Absolute Q integrate partitioning, amplification, and detection within a single instrument, simplifying the workflow and reducing manual handling. These differences may influence laboratory efficiency and operational costs depending on the specific analytical context. While a detailed comparative evaluation of these parameters is beyond the scope of the present study, the observed differences suggest that each platform may offer specific practical advantages depending on laboratory needs, throughput demands, and available resources.

### 3.7. Measurement Uncertainty (MU) Estimation

The evaluation of measurement uncertainty (MU) across the three digital PCR platforms showed a high degree of similarity. This outcome was expected, as the contribution of the instrument itself to the overall uncertainty is relatively minor when compared to other sources that are intrinsic to the method and common to all platforms. Several uncertainty sources are shared across all systems, including those related to the certified reference material (e.g., assigned value and homogeneity), dilution steps, and sample preparation. These factors represent the dominant contributors to the overall MU and are independent of the specific platform used.

In contrast, the components that may vary among platforms are mainly associated with platform-specific features, such as partition generation (droplet-based versus nanoplate-based systems), total reaction volume, number and volume of partitions, and instrument-specific fluorescence detection. However, the impact of these platform-dependent factors on the overall MU was limited and did not result in relevant differences among the evaluated systems. In the case of sample compliance, the MRPL (minimum required performance limit) level was observed for events authorized under Regulation 1829/2003 and for events falling under Regulation 619/2011 [50], and no differences between the digital PCR platforms were observed.

This limited impact can be explained by the fundamental principle of digital PCR, which relies on absolute quantification based on Poisson statistics. In this context, the estimation of target concentration depends primarily on the proportion of positive and negative partitions rather than on amplification efficiency or signal intensity, thereby reducing the influence of platform-specific detection characteristics.

Consequently, the observed similarity in measurement uncertainty across platforms reflects the fact that the analytical principle and quantification model of digital PCR remain the same, regardless of the technological implementation. Therefore, despite the differences in partitioning approaches (droplet-based versus microchamber-based systems), all evaluated platforms (Bio-Rad QX200™, QIAcuity Digital PCR System, and QuantStudio Absolute Q) rely on the same statistical framework, which contributes to the consistency of uncertainty estimates. These findings indicate that, when validated methods and appropriate quality controls are applied, digital PCR platforms provide comparable uncertainty estimates, supporting their interchangeable use for the quantification of the NK603 maize event in compliance with regulatory and methodological requirements [2,31,35,51].

## 4. Conclusions

In this study, three digital PCR (dPCR) platforms were compared for the detection and quantification of the genetically modified maize event NK603 in the duplex PCR assay with the endogenous reference gene (HMG). Overall, the results demonstrate that all evaluated platforms meet the predefined acceptability criteria, including parameters related to trueness, precision, sensitivity and robustness, confirming their suitability for GMO analysis. The performance of the method is particularly relevant in the context of the 0.9% regulatory threshold established in the European Union for GMO labeling. The observed accuracy and precision support reliable discrimination between samples below and above this threshold, which is critical for regulatory compliance and enforcement. However, it should be considered that regulatory thresholds for GMO labeling may vary across different countries and jurisdictions. Therefore, the impact of analytical variability and method performance should be interpreted in relation to the specific regulatory framework in which the method is applied. The specificity of the assay was carefully assessed. Specificity testing was originally performed by the EURL for the NK603 target and the endogenous reference gene (HMG) under singleplex conditions, demonstrating the absence of cross-reactivity and non-specific amplification. The present study confirms that this specificity is maintained when the assay is applied within a digital PCR framework, supporting the reliability of the method for event-specific GMO detection.

Beyond the fact that all platforms fulfilled the analytical performance requirements, the comparison among the three dPCR systems highlights important differences linked to their underlying technological principles. These include the nature of partitioning (microchamber partitions versus droplet-based systems), as well as differences in workflow complexity, reaction volume, time to result, and level of manual handling. Such aspects directly impact the practicability of each platform, particularly in routine laboratory settings.

Operationally, the QuantStudio Absolute Q and QIAcuity Digital PCR System allows more streamlined workflows. Indeed, their microfluidic assay technology, featuring approximately 20,000 uniform reaction wells, ensures consistent sample partitioning and the sealed wells format minimizes the risk of contamination by amplicons or exogenous nucleic acids. The integrated, all-in-one workflow combining sample partitioning, thermal cycling, and fluorescence detection within a single instrument substantially simplifies laboratory procedures, reduces hands-on time, and limits operator-dependent variability. Importantly, the experimental protocol does not require specialized sample handling, allowing users familiar with standard qPCR workflows to adopt digital PCR without additional training. By comparison, the Bio-Rad QX200™ ddPCR platform generates a large number of partitions per sample using a droplet-based approach within a 96-well plate format and requires separate steps for droplet generation and signal reading. These differences reflect distinct technological approaches to digital PCR rather than inherent performance advantages.

At low concentration levels (e.g., 0.1% *m*/*m*), an increase in variability is expected due to the proximity to the detection limit. Although the results remained within the acceptance criteria, this increased variability should be carefully taken into account in laboratory decision-making, particularly when interpreting results close to the limit of quantification or decision threshold. It should be noted that this study was conducted using certified reference materials (CRMs), which represent well-characterized and controlled matrices. In routine analysis, more complex or processed matrices may introduce additional sources of variability, such as DNA degradation or the presence of inhibitors, potentially affecting method performance.

Therefore, while the three digital PCR platforms showed a good agreement and were comparable for the detection and quantification of the NK603 maize event (% *m*/*m*), the choice of platform should not rely solely on performance criteria, but rather on overall fitness for purpose. Platform selection should also take into account operational factors such as ease of use, throughput, hands-on time, and overall laboratory efficiency in line with the minimum performance requirements (MPRs) for GMO analysis. These considerations are essential for informed decision-making when implementing digital PCR technologies in official control and routine testing laboratories, as the cost per analysis may vary across platforms depending not only on consumables and reagents but also on labor and workflow efficiency. Although the present study focused on the NK603 event, the general approach is expected to be applicable to other GM events.

Finally, while the results obtained in this study demonstrate promising performances, a collaborative interlaboratory validation is necessary to further confirm the robustness, reproducibility, and transferability of the method across different laboratories and analytical settings.

## Figures and Tables

**Table 1:** Determination of LOQasym of the NK603 specific dPCR module expressed in copies per reaction (cp/rxn). Outlier evaluation determined by Grubb’s test.

	GM NK603 0.1% *m*/*m*					
	Bio-Rad		QIAcuity		Thermo Fisher	
	GM cp/rxn	Endogeneous gGene cp/rxn	GM cp/rxn	Endogeneous Gene cp/rxn	GM cp/rxn	Endogeneous Gene cp/rxn
**Copy number**	17.20	33,311.00	33.70	59,240.70	24.26	44,695.11
**Measured average (% ** ***m*** **/** ***m*** **)**	0.10		0.11		0.11	
**RSDr (%)**	24.93		24.91		23.08	
**N. outlier**	4		1		0	
**N. rep**	70		20		15	

**Table 2:** Robustness of the HMG/NK603 duplex assay evaluated at 0.1% *m*/*m*. A total of 16 conditions were tested on the Bio-Rad platform and 8 conditions on the Thermo Fisher and Qiagen platforms.

Protocol		NK603								
		Bio-Rad QX200			Qiagen QIAcuity			Thermo Fisher QuantStudio Absolute Q		
		Mean (%)	RSDr (%)	Bias (%)	Mean (%)	RSDr (%)	Bias (%)	Mean (%)	RSDr (%)	Bias (%)
**Ramp rate change and annealing temp Unchange**	Original	0.11	12.23	11.80	0.13	5.60	26.20	0.09	26.94	8.92
	−10% Primer Probe −10% master mix	0.12	13.83	23.26	0.13	7.18	27.53	0.09	20.12	10.52
	−10% Primer Probe	0.12	29.25	22.79	0.12	6.78	24.25	0.09	15.29	8.58
	−10% Master Mix	0.12	19.47	16.24	0.13	5.63	26.77	0.11	16.44	7.79
**Annealing temp +1 °C**	No change PCR reagents	0.11	14.01	14.73	0.12	18.25	21.27	0.10	14.25	1.89
	−10% Primer Probe −10% master mix	0.13	17.64	28.57	0.13	19.72	28.20	0.09	14.14	8.55
	−10% Primer Probe	0.12	21.97	22.38	0.12	18.15	24.81	0.08	18.08	15.23
	−10% Master Mix	0.11	15.18	6.36	0.13	6.15	25.43	0.10	5.10	0.47
**Annealing temp +1 °C and Ramp rate + 0.5 °C/s**	No change PCR reagents	0.11	29.57	8.69	/ ^a^			/ ^a^		
	−10% Primer Probe −10% master mix	0.12	19.47	21.88						
	−10% Primer Probe	0.13	10.29	29.77						
	−10% Master Mix	0.13	13.43	26.43						
**Ramp rate + 0.5 °C/s**	No change PCR reagents	0.13	11.34	27.43	/ ^a^			/ ^a^		
	−10% Primer Probe −10% master mix	0.12	26.80	17.95						
	−10% Primer Probe	0.14	22.58	29.83						
	−10% Master Mix	0.13	6.39	26.13						

^a^ The setting of ramp rate up was not available as option in the QIAcuity and QuantStudio instruments.

**Table 3:** Dynamic range of GM NK603 expressed in GM level percentage (% *m*/*m*) and the values of trueness (Bias %) and precision (RSDr). Samples were analyzed by duplex dPCR.

	NK603								
	Bio-Rad QX200			Qiagen QIAcuity			Thermo Fisher QuantStudio Absolute Q		
GM Level (%)	Measured GM Content (% *m*/*m*)	RSDr (%)	Bias (%)	Measured Mean (%)	RSDr (%)	Bias (%)	Measured Mean (%)	RSDr (%)	Bias (%)
0.1	0.10	24.85	2.47	0.10	20.87	1.33	0.11	22.51	5.45
0.2	0.20	23.50	2.83	0.20	12.36	2.44	0.18	16.42	8.06
0.5	0.46	13.27	5.80	0.45	10.46	7.93	0.48	24.32	1.25
1	1.03	18.31	5.16	1.01	7.96	2.92	1.21	18.63	23.45
2	2.08	10.29	6.24	2.05	9.02	4.83	1.79	13.03	8.80
5	5.29	15.45	7.80	5.48	4.12	9.59	5.12	17.24	2.47

**Table 4:** Linearity GM level duplex assay HMG/GM event (%) using CRM with GM mass fractions ranging from 0.1% to 4.91%.

PCR Run	NK603								
	Bio-Rad QX200			Qiagen QIAcuity			Thermo Fisher QuantStudio Absolute Q		
	Slope	Intercept	R^2^	Slope	Intercept	R^2^	Slope	Intercept	R^2^
R 1	0.79	0.13	1.00	0.86	0.06	1.00	1.05	0.02	1.00
R 2	1.11	−0.05	1.00	0.89	0.07	1.00	0.93	0.04	0.99
R 3	0.90	0.05	1.00	0.92	0.08	0.99	0.96	0.05	0.99
R 4	0.93	0.04	1.00	0.90	0.06	1.00	1.00	−0.01	0.99
R 5	0.94	0.001	1.00	/ ^a^			0.90	0.01	0.99
Mean	0.93	0.05	1.00	0.89	0.07	1.00	0.98	0.02	0.99

^a^ Four runs were performed on the QIAcuity platform.

**Table 5:** Comparison of performance parameters for the duplex dPCR assays for NK603 using Bio-Rad QX200™, Qiagen QIAcuity, and Thermo Fisher QuantStudio Absolute Q platforms.

	Performance Parameters																							
	Bio-Rad QX200								Qiagen QIAcuity								Thermo Fisher QuantStudio Absolute Q							
GM Level (% *m*/*m*)	N Rep	Measured GM Content (% *m*/*m*)	Sr	RSDr %	Ubias	Bias	Bias %	U (%, with k = 2)	N Rep	Measured GM Content (% *m*/*m*)	Sr	RSDr%	Ubias	Bias	Bias %	U (%, with k = 2)	N Rep	Measured GM Content (% *m*/*m*)	Sr	RSDr%	Ubias	Bias	Bias %	U (%, with k = 2)
0.1	30	0.10	0.02	24.85	0.04	0.002	2.47	0.01	24	0.10	0.02	20.87	0.4	0.001	1.33	0.04	15	0.11	0.02	22.51	0.40	0.005	5.45	0.04
0.2	30	0.20	0.05	23.50	0.37	0.006	2.83	0.04	24	0.20	0.02	12.36	0.20	0.01	2.44	0.02	15	0.18	0.03	16.42	0.21	0.016	8.06	0.02
0.5	30	0.46	0.061	13.27	0.51	0.03	5.80	0.05	24	0.45	0.05	10.46	0.51	0.04	7.93	0.05	15	0.48	0.12	24.32	0.59	0.006	1.25	0.07
1	30	1.03	0.19	18.31	0.78	0.05	5.16	0.08	24	1.01	0.08	7.96	0.72	0.03	2.92	0.07	15	1.21	0.23	18.63	0.91	0.230	23.45	0.10
2	30	2.08	0.21	10.29	0.98	0.12	6.24	0.10	24	2.05	0.19	9.02	0.98	0.09	4.83	0.11	15	1.79	0.23	13.03	1.08	0.172	8.80	0.13
5	30	5.29	0.818	15.45	2.03	0.38	7.80	0.25	24	5.48	0.23	4.12	1.42	0.48	9.59	0.15	15	5.12	0.88	17.24	2.64	0.124	2.47	0.35

## Data Availability

The original contributions presented in this study are included in the article/Supplementary Materials. Further inquiries can be directed to the corresponding author.

## Supplementary Materials

The following supporting information can be downloaded at: https://www.mdpi.com/article/10.3390/foods15081366/s1, Figure S1: Optimization of primer and probe concentrations for the NK603 digital PCR assay; Figure S2: Description of the calculation approach; Figure S3: High specificity of both primer/probe systems; Table S1: Primer and probe; Table S2: Specificity.
