# Supplementary material for: Validation of a Duplex Digital PCR Assay for the Quantification of the NK603 Maize Event Across Three dPCR Platforms
Source: Foods. 2026 Apr 14;15(8):1366. doi: 10.3390/foods15081366 (PMC13114549; doi:10.3390/foods15081366)
Supplement: Supplementary file 1 [file foods-15-01366-s001.zip › Figure S2.pdf]

The uncertainty was determined, firstly calculating the GM mass fraction (wRM) of the reference material prepared in laboratory following the formula describing in Verginelli et al (2024)[20] ;

$$w_{\mathrm{RM}} = \frac{c_{\mathrm{CRM}} \times v_{\mathrm{CRM}} \times w_{\mathrm{GM,CRM}}}{c_{\mathrm{NGM}} \times v_{\mathrm{NGM}} + c_{\mathrm{CRM}} \times v_{\mathrm{CRM}}}$$

where:

w_{RM}: GM mass fraction of the prepared reference material (in g/kg)

c_{CRM} : concentration (in copies/µL) of the *hmg* target in the DNA solution extracted from the CRM

v_{CRM} : volume (in µL) taken of the DNA solution extracted from the CRM

$w_{\mathrm{GM,CRM}}$: certified value for the GM mass fraction of the CRM (in g/kg)

c_{NGM} : concentration (in copies/µL) of the *hmg* target in the DNA solution extracted from the NGM

v_{NGM} : volume (in µL) taken of the DNA solution extracted from the NGM

Secondly in the next step we estimated the uncertainty associated with the produced reference material considering the individual standard uncertainty contributions for each variable, as described in Trapmann et al (2014)[37] and in Verginelli et al (2024)[20]:

- uncertainty of the HMG concentration of the DNA solution extracted from the CRM (uc,CRM): estimated as standard error of the mean of cCRM;
- uncertainty of the HMG concentration of the DNA solution from the non-GM material (uc,NGM): estimated as standard error of the mean of cNGM;
- uncertainty associated with the certified value of the CRM used (uw,CRM): standard uncertainty of the certified value taken;
- uncertainty associated with the volume of the taken GM DNA solution (uv,CRM): estimated as standard error of the pipette volume taken from the technical specification of the pipette;
- uncertainty associated with the volume of the taken NGM DNA solution (uv,NGM): estimated as standard error of the pipette volume taken from the technical specification of the pipette.

The combined uncertainty was estimated considering the determination of DNA concentration, the pipetting, the volume mixed and the purity of materials. It was calculated by taking the square root of the sum of squares of these values following the formula[37];

$$u = \sqrt{u_{(wGM,mGM)}^{2} + u_{(wGM,pGM)}^{2} + u_{(wGM,mNGM)}^{2} + u_{(wGM,ipNGM)}^{2}}$$

u	combined standard uncertainty
$u_{(wGM,\ mGM)}$	standard uncertainty in function of w_{GM} and the weighing GM material
$u_{(wGM,\ pGM)}$	standard uncertainty in function of w_{GM} and the purity GM material
$u_{(wGM,\ mNGM}$	standard uncertainty in function of w_{GM} and the weighing non-GM material
$u_{(wGM,\ ipGM)}$	standard uncertainty in function of w_{GM} and the impurity non-GM material
