# Supplementary material for: Validation of a Duplex Digital PCR Assay for the Quantification of the NK603 Maize Event Across Three dPCR Platforms
Source: Foods. 2026 Apr 14;15(8):1366. doi: 10.3390/foods15081366 (PMC13114549; doi:10.3390/foods15081366)
Supplement: Supplementary file 1 [file foods-15-01366-s001.zip › Figure S3.pdf]

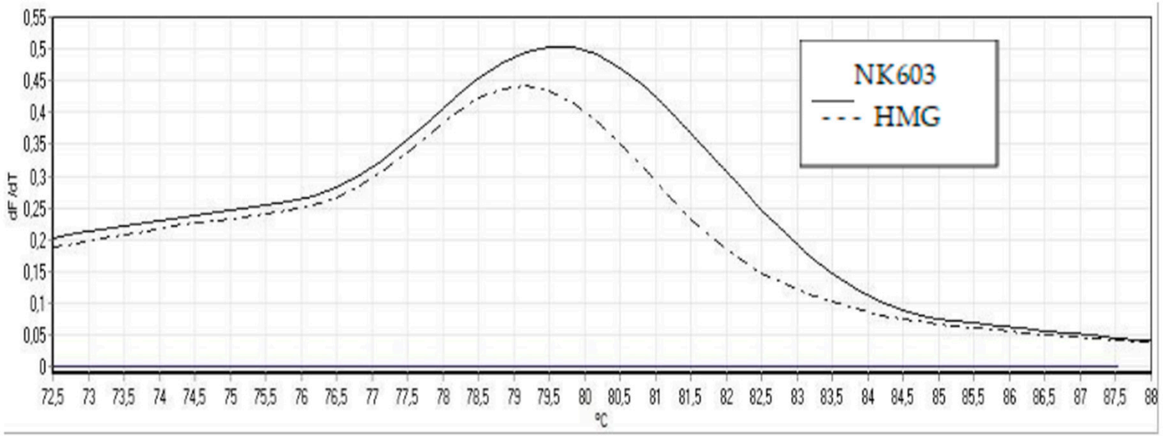

Figure S3. Melt curves of the primers used in the duplex assay for NK603 and HMG. The melting curve analysis was performed to verify amplification specificity and to detect the presence of potential non-specific products or primer–dimer formations.
