# Supplementary material for: Validation of a Duplex Digital PCR Assay for the Quantification of the NK603 Maize Event Across Three dPCR Platforms
Source: Foods. 2026 Apr 14;15(8):1366. doi: 10.3390/foods15081366 (PMC13114549; doi:10.3390/foods15081366)
Supplement: Supplementary file 1 [file foods-15-01366-s001.zip › Table S1.pdf]

Table S1. Specifications of primer and probe system.

Target	Primer and probe	Gene name and Sequence 5'–3'	Final concentration			URL
			**qPCR pmol/µl**	**ddPCR QX200 (Bio-Rad) and dPCR QuantStudio Asolute Q (Thermo Fisher) pmol/µl**	**dPCR QIAcuity (Qiagen) pmol/µl**	
NK603	NK603 primer F	5’- ATg AAT gAC CTC gAg TAA gCT TgT TAA-3’	0.15	0.6	0.8	QT-EVE-ZM-008 https://gmo-crl.jrc.ec.europa.eu/summaries/NK603-WEB-Protocol%20Validation.pdf
	NK603 primer R	5’- AAg AgA TAA CAg gAT CCA CTC AAA CAC T –3’	0.15	0.6	0.8	
	NK603 probe	6-FAM 5'- Tgg TAC CAC gCg ACA CAC TTC CAC TC- 3’ BHQ1	0.05	0.25	0.4	
HMG	MaiJ-F2	5’ -TTg gAC TAg AAA TCT CgT gCT gA –3’	0.15	0.6	0.8	http://gmo-crl.jrc.ec.europa.eu/gmomethods/entry?db=gmometh&id=qt-tax-zm-002&q=QT-TAX-ZM-002
	mhmg -rev	5’ -gCT ACA TAg ggA gCC TTg TCC T –3’	0.15	0.6	0.8	
	Mhmg -probe	HEX 5’-CAA TCC ACA CAA ACg CAC gCg TA-3’BHQ	0.05	0.25	0.4	
