# Supplementary material for: Validation of a Duplex Digital PCR Assay for the Quantification of the NK603 Maize Event Across Three dPCR Platforms
Source: Foods. 2026 Apr 14;15(8):1366. doi: 10.3390/foods15081366 (PMC13114549; doi:10.3390/foods15081366)
Supplement: Supplementary file 1 [file foods-15-01366-s001.zip › Table S2.pdf]

Table S2. A) Outcome of self-dimer evaluation by Primer-dimer software B) Outcome of cross-dimer evaluation by Oligo-evaluator software.

A

Forward Primer Name	Forward Primer Seq	Reverse Primer Name	Reverse Primer Seq	Structure	DG
NK603 F	ATGAATGACCTCGAGTAAGCTTGTTAA	NK603 R	AAGAGATAACAGGATCCACTCAAACACT	heterodimer	-2.89
NK603 F	ATGAATGACCTCGAGTAAGCTTGTTAA	NK603 P	TGGTACCACGCGACACACTTCCACTC	heterodimer	-0.24
NK60 3R	AAGAGATAACAGGATCCACTCAAACACT	NK603 P	TGGTACCACGCGACACACTTCCACTC	heterodimer	0
MaiJ-F2	TTGGACTAGAAATCTCGTGCTGA	Mhmg-probe	CAATCCACACAAACGCACGCGTA	heterodimer	-8.12
mhmg-rev	GCTACATAGGGAGCCTTGTCCT	Mhmg-probe	CAATCCACACAAACGCACGCGTA	heterodimer	-8.12
MaiJ-F2	TTGGACTAGAAATCTCGTGCTGA	mhmg-rev	GCTACATAGGGAGCCTTGTCCT	heterodimer	-0.01
MaiJ-F2	TTGGACTAGAAATCTCGTGCTGA	NK603 R	AAGAGATAACAGGATCCACTCAAACACT	heterodimer	0
NK603 F	ATGAATGACCTCGAGTAAGCTTGTTAA	mhmg-rev	GCTACATAGGGAGCCTTGTCCT	heterodimer	-0.2
NK603 F	ATGAATGACCTCGAGTAAGCTTGTTAA	Mhmg-probe	CAATCCACACAAACGCACGCGTA	heterodimer	-8.12

B

Name	Sequence	Base Count	Length (bp)	Molecular Weight	Tm (°C)	Extinction Coefficient	µg/OD at 260 nm	GC%	GC Clamp	Run Length (bp)	Primer Dimer	Secondary Structure
NK603 F	ATGAATGACCTCGAGTAAGCTTGTTAA	A = 9, U = 0, G = 6, C = 4, T = 8, I = 0, Total = 27	27	8322.5	65.6	272.8	30.5	37.0	1	2	Yes	None
NK603 R	AAGAGATAACAGGATCCACTCAAACACT	A = 13, U = 0, G = 4, C = 7, T = 4, I = 0, Total = 28	28	8567.7	65.9	289.1	29.6	39.3	1	3	No	Weak
NK603 P	TGGTACCACGCGACACACTTCCACTC	A = 6, U = 0, G = 4, C = 11, T = 5, I = 0, Total = 26	26	7836.2	75.4	236.4	33.1	57.7	1	2	No	Weak
MaiJ-F2	TTGGACTAGAAATCTCGTGCTGA	A = 6, U = 0, G = 6, C = 4, T = 7, I = 0, Total = 23	23	7078.7	64.9	224.0	31.6	43.5	2	3	No	None
Mhmg-rev	GCTACATAGGGAGCCTTGTCCT	A = 4, U = 0, G = 6, C = 6, T = 6, I = 0, Total = 22	22	6726.5	64.5	205.4	32.7	54.5	2	3	No	Weak
Mhmg-probe	CAATCCACACAAACGCACGCGTA	A = 9, U = 0, G = 3, C = 9, T = 2, I = 0, Total = 23	23	6955.6	73.6	224.9	30.9	52.2	3	3	No	Weak
