# Supplementary figures and images for: Validation of a Duplex Digital PCR Assay for the Quantification of the NK603 Maize Event Across Three dPCR Platforms
Source: Foods. 2026 Apr 14;15(8):1366. doi: 10.3390/foods15081366 (PMC13114549; doi:10.3390/foods15081366)

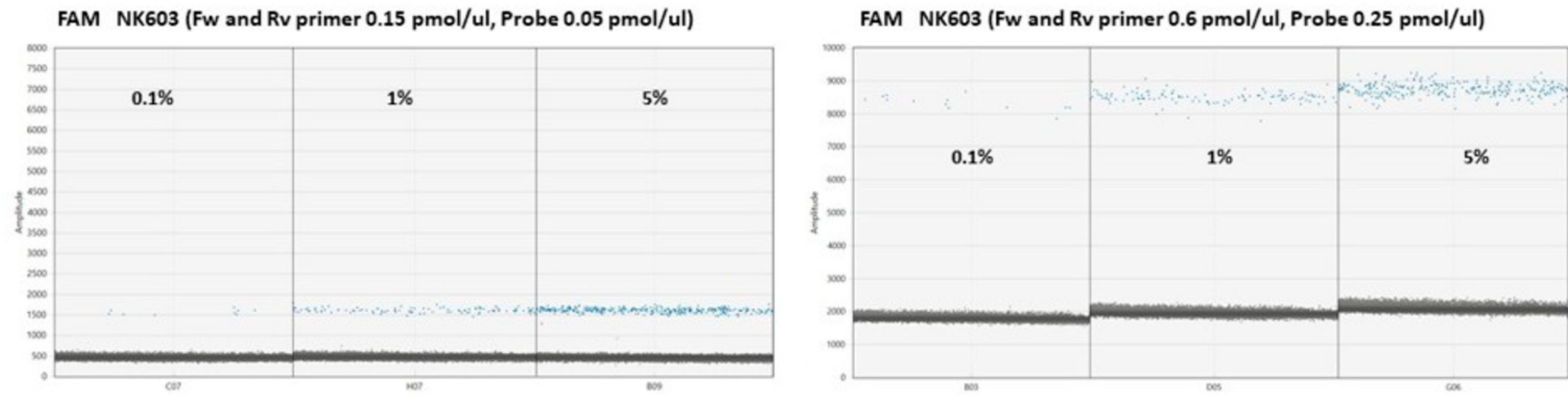

**Figure S1.** Optimization of primer and probe concentrations for the NK603 digital PCR assay.

Supplement: Supplementary file 1 [file foods-15-01366-s001.zip › Figure S1.pdf]
